# Older adults in social housing: A systemically vulnerable population that needs to be prioritized

**DOI:** 10.1093/haschl/qxae154

**Published:** 2024-11-27

**Authors:** Jasmine Dzerounian, Guneet Mahal, Leena AlShenaiber, Ricardo Angeles, Francine Marzanek, Melissa Pirrie, Gina Agarwal

**Affiliations:** Department of Family Medicine, McMaster University, 100 Main St W, Hamilton, Ontario L8P 1H6, Canada; Department of Health Research Methods, Evidence, and Impact, McMaster University, 1280 Main St W, Hamilton, Ontario L8S 4L8, Canada; Department of Family Medicine, McMaster University, 100 Main St W, Hamilton, Ontario L8P 1H6, Canada; Department of Family Medicine, McMaster University, 100 Main St W, Hamilton, Ontario L8P 1H6, Canada; Department of Health Research Methods, Evidence, and Impact, McMaster University, 1280 Main St W, Hamilton, Ontario L8S 4L8, Canada; Department of Family Medicine, McMaster University, 100 Main St W, Hamilton, Ontario L8P 1H6, Canada; Department of Family Medicine, McMaster University, 100 Main St W, Hamilton, Ontario L8P 1H6, Canada; Department of Family Medicine, McMaster University, 100 Main St W, Hamilton, Ontario L8P 1H6, Canada; Department of Family Medicine, McMaster University, 100 Main St W, Hamilton, Ontario L8P 1H6, Canada; Department of Health Research Methods, Evidence, and Impact, McMaster University, 1280 Main St W, Hamilton, Ontario L8S 4L8, Canada

**Keywords:** older adults, social housing, underserved population, vulnerable population, social determinants of health, policy

## Abstract

Older adults living in social housing are a vulnerable population with unique health challenges that often lead to poor health outcomes and high emergency service utilization. However, the needs of this population are frequently overlooked. This policy note describes the characteristics of older adults living in social housing in Canada and discusses why they are a vulnerable, underserved population in need of immediate attention and priority. Older adults in social housing have higher rates of chronic disease, lower quality of life, and lower health literacy and face challenges caused by various compounding social determinants of health. There is a large gap in research and tailored interventions focusing on this population. Based on these findings, the authors highlight the need for the allocation of resources to support this growing population, including dedicated funding, research, and programming. Proactively addressing the issues that exist in the health and social care of this high-needs population will also have larger implications for reducing healthcare system burden.

## Teaser

Older adults living in social housing are a highly vulnerable population. Chronic disease, poor mental health, poverty, and social isolation are more highly prevalent among this group, and the health needs of this population frequently remain unmet. This creates a cascade of negative health outcomes, leading to situations of health crisis and potential for high reliance on emergency systems. This is why older adults in social housing need immediate prioritization from researchers, governments, and health systems.

## What is social housing?

Social housing, also known as community or public housing, is lower-cost rental housing provided by government, regional, or not-for-profit organizations.^1^ It is designed to serve low-income individuals who cannot afford housing in the private rental market.^2^ There are many types of social housing programs that exist, including purpose-built low-income rentals, rent-geared-to-income units, subsidized units in market-rate buildings, and market-rate apartments that are partially funded by governmental rent supplements.^3^ Social housing is open to different types of people who may be in need of these programs, for example, single adults, families with children, or older adults living with low income.^4^

## Lack of research on older adults in social housing

Older adults living in social housing are a highly vulnerable population, but published literature on their health needs is scarce. This is due to the many barriers that affect their engagement in research, due in part to specific social determinants of health. For example, individuals living in social housing generally have low education, low literacy, limited Internet access, and other challenges that make traditional surveys unfeasible for them to complete.^5,6^ As a result, this population is harder to reach and more inaccessible to researchers, creating difficulties in generating knowledge about older adults living in social housing. Furthermore, this inaccessibility to older adults in social housing through traditional research methods makes it even more challenging to incorporate their voices into identifying research gaps to later influence policy development. As such, very little is known about older adults in social housing and their needs are therefore largely overlooked.

## A vulnerable population

An emerging body of literature has begun to more clearly illustrate the unique and numerous challenges that residents of social housing face compared with the general population. Many individuals with low income live in social housing because they need housing that is adjusted to their level of income. Moreover, residents of social housing face the additional challenges associated with living with low income. Poverty and housing are both established social determinants of health. These can compound as barriers to accessing healthcare or resources, quality education, and safe and secure living conditions.

For residents of social housing, low income and some of the living conditions of the built environment within social housing (for example, the condition of buildings they live in, neighborhoods, or nearby spaces like parks and schools) can pose a risk to their health.^7–9^ As of 2023, nearly 70% of social housing buildings in Canada were built prior to 1986,^10^ the majority of which are present in urban neighborhoods.^11^ Rural areas have an uneven distribution of and limited social housing buildings.^12^ Based on data from the Canadian Mortgage and Housing Corporation, approximately one-third of all social housing buildings are in below-average “fair” or “poor” conditions.^10^ A prominent concern among people living in social housing buildings is feeling unsafe and fear of crime in their community and neighborhood.^9^ In urban areas, people living in social housing have access to services (such as food supports and crisis interventions), but older adults have difficulty in accessing/utilizing these supports.^13^ In contrast, there is a lack of services available in rural areas, particularly with regard to people requiring crisis services.^12^

As a population, the majority of older adults in social housing are women and persons living alone.^14^ They also experience a lower measured and self-reported quality of life and low health literacy.^6,14^ In recent years, especially with the increase in the usage of virtual support services, digital literacy and access have become an important social determinant of health.^15^ However, older adults in social housing have limited access to technology and low digital literacy, lacking practical skills, digital confidence, and financial support.^16,17^ This is a unique population and therefore may require interventions specific to their demographics that address their determinants of health with the goal of increasing their quality of life. The challenges residents of social housing face because of social determinants of health culminate in a variety of adverse health outcomes. Chronic disease, poor mental health, poverty, and social isolation are more prevalent among low-income individuals, many of whom live in social housing.^18–22^

One Canadian multisite intervention conducted in subsidized housing for older adults in 5 Ontario communities indicated, by self-reported data, that 27.4% of participants had diabetes, 54.4% had hypertension, and 48.7% reported anxiety and/or depression.^5^ Participants also had poor health literacy and challenges with mobility, which can limit access to health education/prevention programs and primary care.^5,6^ It is important to note that this study considered self-reported data only.

### Chronic disease rates and risk factors

Further research has corroborated and expanded upon these findings. For example, one study of older adults (ages 55+) living in Ontario social housing found that 96.7% of low-income older adults had a moderate to high risk of developing diabetes based on lifestyle-related modifiable risk factor assessments. This study further concluded that undiagnosed prediabetes and diabetes among older adults in social housing in Ontario are likely close to 32.0%.^23^ Moreover, in the same population, the majority of older adults in social housing have either undiagnosed or unmanaged high blood pressure, as found by cardiovascular disease and diabetes risk assessments using validated questionnaires and automated blood pressure readings. There is also a high prevalence of modifiable risk factors for high blood pressure, including but not limited to smoking, low fruit and vegetable intake, and low physical activity. Primary care interventions appropriate for this population can have protective effects against future cardiometabolic complications, such as coronary heart disease, stroke, or diabetes.^24^

### Poverty, food insecurity, and fall risk

Older adults in social housing also face unique challenges with the risk and fear of falling, which appears to be linked to poverty and food insecurity. Daily fruit and vegetable consumption has been identified as a modifiable risk factor that is protective against a history of falling.^25^ However, older adults living in social housing experience food insecurity at approximately twice the rate of older adults in Canada's general population, which is related to inaccessibility and increased barriers to healthy foods, including fruits and vegetables. For those older adults in social housing who reported being food secure, their diet was considered to be poor; 42.7% reported eating high-fat or fast foods 1 to 2 times per week, and 33.4% reported that they did not eat at least one serving of fruits and/or vegetables daily.^26^ Furthermore, nutritional risk has been established as a risk factor for an increased risk of falls, particularly in frail older adults.^27^ Having a poor diet is also linked to the development of cardiovascular disease,^24^ which increases the need for blood pressure medications and the risk of stroke, both of which are established risk factors for falls.^25^ Vulnerable populations such as older adults in social housing already have poor access to healthcare, which means that they are not able to address their health-related risk factors for falls. Besides the implications of poor diet for the development of chronic disease, these subsequent research findings suggest that older adults in social housing are at systemic risk of falling as an effect of food insecurity and poverty.

### Social isolation and loneliness

In addition to chronic disease, risk of falling, and food insecurity, older adults in social housing are at risk of facing adverse health outcomes associated with social isolation and loneliness. Social isolation and loneliness are largely unexplored in older adults living in social housing specifically; however, many characteristics present among older adults in social housing place them at higher risk for social isolation and loneliness than the general population. As mentioned above, social isolation is prevalent among low-income individuals, many of whom live in social housing.^21^ Living alone, multiple chronic health problems, and mental health issues are all identified as risk factors for social isolation of older adults in Canada and are all prevalent conditions among residents in social housing, including older adults.^9,14,18,21,23,25^

A study on isolation and social participation in Canadian older adults showed that women were more likely than men to report subjective isolation (a measure of loneliness and community belonging). Lower household education was associated with low social participation among women and subjective isolation for men.^28^ Low social participation was significantly associated with mortality for both men and women. Critically, women who participated less frequently in community-related activities lived ∼8 months less than women who were high participators.^28^ The majority of older adults in social housing are women (72%-80%) and have low levels of formal education, with 65%-75% reporting completion of high school or less.^5,14^ These findings suggest that older adults in social housing are not only at higher risk of social isolation and its adverse health effects due to income and health issues, but also due to demographic factors such as education level and gender.^5,14^

In one American study examining loneliness in homeless-experienced older adults, participants described their loneliness experience in one of 3 ways: they were lonely and distressed due to impairment (eg, mobility challenges and hearing loss) and isolation; they would rather be isolated due to trauma; or they were experiencing transient loneliness due to life experiences associated with aging and grief.^29^ This study shows that loneliness is a complex and layered topic. Older adults in social housing may have unique experiences that influence feelings of loneliness in their lives, and it is a topic that warrants further research.

## Learnings from the pandemic

The Public Health Agency of Canada has reported that the highest inequalities in mortality due to COVID-19 were in residents of low-income areas, apartments, and large urban centers.^30^ People in social housing characteristically fall into these 3 groups.^31^ As well, during the pandemic, seniors were most likely to be hospitalized and admitted to the ICU.^32^ In the first 15 months of the pandemic in Canada, older adults accounted for 93% of deaths caused by the virus.^33^ While older adults in social housing are generally twice as likely to be socially isolated compared with the general population, COVID-19 had increased the risk of social isolation for all older adults.^34,35^

The pandemic has highlighted existing disparities in healthcare. These data expressly underline the high vulnerability of older adults in social housing. Despite this, there are limited healthcare supports tailored to this group.^36^ For example, during the pandemic, there was a focus on public health measures for seniors in long-term care, but older adults in social housing were not adequately supported.^37^ It is important to note that in Ontario there are nearly the same number of seniors in social housing as there are in long-term care.^36^

## Tailored intervention

As emphasized by the impacts of the pandemic, older adults in social housing are a large, vulnerable group and need programs and resources that are targeted to their unique needs. One example of such is the CP@clinic Program, a chronic disease prevention and health promotion program, that has been implemented in social housing buildings. This program facilitated social relationships between older adults and allowed some participants to feel more socially connected to other residents.^38^ During the COVID-19 pandemic, it was also implemented as a telephone adaptation. In this high-needs population, CP@clinic has also been found to improve health and quality of life and reduce 911 calls among participants. Considering these positive outcomes, it is evident that older adults in social housing would greatly benefit from interventions that are more precisely tailored to their unique needs. However, older adults in social housing still lack access to support services. As well, some data show that there is low utilization of existing supports, which suggests that those resources may not be adequate, easily available, or known to residents.^13^

## Research gaps on healthcare utilization

Very little research has characterized health service utilization among high-needs individuals living in social housing. One study in Manitoba examined individuals on the waitlist for subsidized housing with respect to their health service utilization for 1 year pre- and post-move into subsidized housing. The results indicated a significant decrease in utilization during the initial months post-move, though the decrease was not sustained for Emergency Department (ED) visits or family doctor visits, and utilization returned to pre-move levels.^39^ This study was limited to new social housing residents, so it is possible that they had more active referrals to community programs than long-term residents had, and/or had not been exposed to a social housing environment for a significant length of time. This study reflects short-term impact rather than providing the full characterization of health and health service utilization in social housing. Furthermore, it did not look at older adults specifically whose health needs may differ from those of the general population in social housing.

As a result of the scarce literature on this subject, very little is known about health service utilization specific to low-income individuals in social housing. However, a study of EMS calls in Canada showed that 48% of EMS calls came from older adults, 57% of which were women, and many of which presented with falls, or cardiovascular issues.^40^ Older adults in social housing are not only included among those who call 911 most often due to age, but they are also mostly women, and are vulnerable to a risk of falling and cardiovascular disease. Given their additional vulnerability to chronic disease, social isolation, mental health challenges, and low health literacy, older adults in social housing have many healthcare needs. As such, older adults in social housing likely need to access acute care such as costly EMS calls and ED visits due to the health-related inequalities they face. This use of EMS and ED services continues to place a burden on paramedic and emergency services in Ontario, as ED use is outpacing population growth, and older adults continue to increase as a percentage of all ED visits.^41^

## A universal concern

These patterns of vulnerability within this population can be seen worldwide. A cross-sectional study conducted on older adult social housing residents in New York in the United States reported high levels of anxiety, depression, medical comorbidity, and functional impairment.^42^ Furthermore, of those residents with a mental health need, less than half had received treatment.^42^ An Australian study that surveyed social housing tenants revealed that social housing residents had poorer health and higher health risks when compared with the general population, and even socioeconomically disadvantaged people in the same community.^43^ Another study in Singapore found that older adults in social housing had very low life satisfaction, due to factors such as housing conditions, social inclusion, and mobility challenges.^44^ These are just some examples that illustrate the high vulnerability of older adults in social housing around the world. With countries experiencing aging populations^45^ and increasing poverty rates,^46^ it is now more important than ever to address the vulnerabilities and unmet needs of older adults in social housing.

## Conclusion and implications for policy

Older adults in social housing are a large group of vulnerable individuals who are often systematically overlooked. This population is affected by chronic illness, high disease risk, social isolation, poor quality of life, food insecurity, low socioeconomic status, housing concerns, and other social determinants of health.

Despite this need, there are not enough resources allocated to support this growing population. This support could involve dedicated funding, research, and program/resource allocation. There needs to be additional research into the specific characteristics, health status, and healthcare utilization of older adults in social housing. In addition, a care model that offers access to preventive healthcare and that is informed by the demographics, social determinants of health, and needs of this population can address their healthcare use and their particular vulnerabilities. Such a care model has the potential to make a significant impact on the health of older adults in social housing. However, these research and program initiatives can only be established and sustained with the allocation of appropriate community healthcare resources.

By addressing the issues that exist in the health and social care of high-needs populations, we can also reduce burden on and improve the state of our emergency healthcare system. At a time when this system is overwhelmed, and the population is rapidly aging, this is important now more than ever. Extra consideration, support, and priority must be given to older adults in social housing to prevent the continuation of their systemic vulnerability.

## Supplementary Material

qxae154_Supplementary_Data
